# The Editor's Letter-Box

**Published:** 1914-04-25

**Authors:** 


*To the Editor of* THE HOSPITAL.

SIR,—"Interested" seems rather confused. First, he—or she—points out that the statement that a married employee can be dismissed if her employer be dissatisfied shirks the issue because the latter cannot know whether the home be neglected. This implies that "Interested" considers that it is so essentially the duty of an employer to discover whether a married woman employee neglects her home that, as he is absolutely unable to carry out this inquisition, he should dismiss all married women on the "off chance" that one may be treating her family in a way of which he does not approve. The only logical conclusion to such a theory is that all female labour outside the home should be dispensed with.

Probably the unmarried have dependant sisters and mothers, and these may be languishing unattended. Then why exclude men from the sway of this beneficent autocracy? They perchance do not spend a sufficient amount on their families.

The truly conscientious employer should see to it that no married man is given work who will not allow a certain proportion of his weekly wage to be handed direct to the queen of the home, who has been discharged for accepting the onerous, if sometimes honorary, position.

Secondly, he proceeds as follows speaking of the London County Council as a special employer: "It merely, so I judge, regards the enforced absences . . . as prejudicial to the efficiency of school inspection." Quite so. If the enforced absences due to pregnancy, etc., are more detrimental to the efficiency of the service than constant changes in the so-called "permanent staff" get rid of the individuals concerned.

The injustice that we object to is the penalisation of all on account of hypothetical cases. The work of school inspecting is carried out under very similar conditions to that of the married teachers. Their excellent services are a matter of common knowledge. It is difficult to see why all that has been urged in their favour should not be said were married inspectors given a chance.

As to your correspondent's touching belief in the high idea which the Council holds of marriage as "a calling" ("trade" describes it better if one is absolutely dependent on it as a means of livelihood), may I point out that these pious opinions are of very little moment to the victim who receives the same treatment for following "the higher call" as her sister who is turned out for "bad behaviour"?—Yours faithfully,

A WOMAN GENERAL PRACTITIONER.

April 19, 1914.

